# Bilateral leadership in critical moments: France, Germany, and the management of major European integration crises

**DOI:** 10.1057/s41295-023-00344-6

**Published:** 2023-02-15

**Authors:** Lucas Schramm

**Affiliations:** Chair of Political Systems and European Integration, Geschwister-Scholl-Institute of Political Science, Ludwig-Maximialians-University Munich, Oettingenstraße 67, 80538 Munich, Germany

**Keywords:** Bilateralism, Crisis, European integration, France, Germany, Leadership

## Abstract

Not least in view of the past decade, crises in the European Union (EU) have attracted much scholarly attention. At the same time, difficult decision-making situations and turning points have always been parts of the European integration process. Moreover, as founding and the two largest member states, France and Germany have been key drivers in the development of today’s EU polity. Strikingly, a systematic analysis of *major* crises covering the *entire* integration process and the *comparative* role that France–Germany have played, so far is lacking. Scholars tend to focus on instances of ‘successful’ crisis resolution or, more recently, on a presumably hegemonic Germany. This article, by contrast, argues and demonstrates why and how France and Germany, *together*, have been essential for the management and resolution of European-level political controversies and deadlock. To do so, the article considers nine major integration crises. It highlights different ways and means of bilateral leadership and their resulting impact on European integration. Case selection includes both successful crisis management and instances of failed or absent leadership.

## Introduction

‘Crises’ appear to have become a permanent feature of European Union (EU) politics and research. Over the past decade, scholars have analyzed the EU’s most recent challenges including the Euro crisis, migration crisis, and coronavirus pandemic. Crises are particular moments in the development and future direction of a political system because they confront decision-makers with strong alternatives (Boin et al. 2017). Member states and national governments usually are the most important actors with respect to EU crisis management. Due to their (material) capacities and political authority, they have the necessary means to take and implement far-reaching decisions (Van Middelaar 2019). Primarily because of the Euro crisis one decade ago, and thanks to its large financial resources, scholars have predominantly focused on the role of Germany (Bulmer and Paterson 2019).

By contrast, this article shows that crises have been an important part of the European integration process since the beginning. Some scholars tend to neglect that European integration actually started with a major setback, when member states failed to implement a supranational army in the context of the European Defence Community (EDC). Moreover, the article holds that France and Germany, together, have been indispensable for EU crisis management and resolution. As an “anti-hegemonic” enterprise avoiding disastrous power politics, and in situations with varying actor constellations and policies, leadership during EU crises usually comes as shared or joint leadership (Schild 2010).

The article argues and demonstrates that France–Germany exercise different ways of crisis leadership. These notably include (a) diplomatic brokerage and putting together package deals which tend to represent larger camps of member states, and (b) carrying financial burdens that are necessary for crisis resolution. Such successful leadership either displays a complementary and additive logic, with both France and Germany realizing key national preferences, or they follow a convergent perspective in which France–Germany agree on joint crisis instruments (Krotz and Schild 2013). However, going beyond accounts of ‘successful’ crisis management only, the article also considers cases where Franco–German leadership did not emerge in the first place, or failed to make a significant impact on the EU level. Such latter instances were due to (a) ‘domestic politics’ and ratification problems in at least one of the two countries, with Franco–German relations themselves often being the subject of major controversies, or (b) lacking common vision and objective for crisis resolution on the part of France and Germany. In practice, in the individual cases, combinations of the complementary logic and the convergent perspective, as well as of ‘domestic politics’ and lacking objectives for crisis resolution, are possible.

An important implication of this analytical perspective is that the logic of Franco–German bilateralism and the potential for Franco–German leadership have been remarkably constant over time. This holds for both electoral and party-political changes in government in the two countries and for developments at the European level, such as an increase in the number of member states following various enlargement rounds. Of course, Franco–German leadership in an EU of 28 (or 27) member states differs from the European Economic Community (EEC) with six member states some 60 years ago. Also, an economically stronger, fully sovereign, and more populous Germany, following the country’s unification in 1990, had increased bargaining clout during European negotiations. At the same time, European negotiations, and German bargaining power in particular, are context and policy-field specific. Moreover, the potential for bilateral leadership, when France and Germany represent different camps of member states and jointly initiate policy proposals, still applies in a larger and supposedly more heterogeneous EU.

The article is structured as follows. The next section assesses the prospects of Franco–German EU leadership during crises. It highlights why, in times of great uncertainty, France–Germany, rather than other member states or the EU’s supranational institutions, are the central actors to provide guidance and offer policy solutions. The empirical part first looks at five instances of successful Franco–German crisis management, before analyzing four cases of absent or failed Franco–German leadership. These sections highlight different ways and means of bilateral leadership and the conditions for France–Germany to make a decisive impact on EU-level decision-making. The conclusions summarize the main findings, stressing theoretical and empirical implications of this study and suggesting avenues for future research.

## Franco–German leadership in crisis times

Leadership implies certain political actors providing stability to a political system and giving guidance for other actors. Stability and guidance come via the definition of common objectives, the development of joint policy proposals, and determined political action for their realization (Kindleberger 1973). Leadership further requires actors who are able and willing to make use of their indispensable power resources. While ability refers to the presence of material, institutional, and/or diplomatic resources, willingness presupposes the capacity of the political personnel, both ‘at home’ and among fellow governments, to assume a prominent role (Schoeller 2019). Crises, in particular, require political leadership. As decisive moments threatening key features of the EU polity, they call for fast decisions against the background of great uncertainty (Boin et al. 2017).

In the EU, leadership during crises primarily comes from the member states. Other than the EU’s supranational actors like the European Commission or the European Parliament, member states usually have available the necessary resources, such as money and large administrative and diplomatic capacities, to work out and realize political compromises. This, in turn, puts national executives center stage, who have the authority to take and implement far-reaching decisions (Van Middelaar 2019). Moreover, with respect to the EU, one actor permanently taking the lead is unlikely as such a predominant position would meet the opposition of other member states. Leadership in the EU therefore usually takes the form of shared or joint leadership among a limited group of member states (Schild 2010).

Numerous scholars have stressed the pivotal role that France and Germany, both on their own but even more so when acting together, have played in shaping today’s EU polity. Accounts of (lacking) Franco–German leadership date back to the 1950s and cover every decade of European integration. Writing in the 1960s, Roy Willis (1968) remarked that both countries, due to their size and the importance they attach to European integration given their conflict-ridden past, act as “focal points” for other member states. Another long-time observer noted that “no relationship has been more important to the development of the integration process than that between France and Germany”. This might be because of their large bilateral resources and/or because no alternative group of member states has similar resources at their disposal (Cole 2001: vii, 146–147). At the end of the last century, Douglas Webber (1999) noted the remarkable influence that France–Germany exercise across different EU policy fields. Somewhat counterintuitively, at first sight, he argued that the potential for Franco–German leadership in the EU was greatest if the two countries shared a common vision for how to overcome a given problem but started from different positions since such a constellation opens up opportunities for package deals among all, or most, member states.

However, little theoretical and analytical work has appeared on the conditions and precise forms of Franco–German leadership, especially during crisis times. Moreover, scholars so far have predominantly focused on ‘successful’ instances of Franco–German leadership. With respect to analytical categories, Krotz and Schild (2013) suggest different forms of Franco–German leadership in European integration and EU politics ranging from the pursuit of deeper integration to the EU’s representation in foreign affairs, to the management of crises. These forms, in turn, materialize through different ways including setting the political agenda, building compromises and consensus—first between France–Germany themselves and then on the European level—and forging coalitions of like-minded member states. However, also these authors do not further define the precise means of Franco–German EU crisis management, let alone the conditions for the emergence and potential impact of bilateral leadership during European integration crises. Regarding case selection, scholars usually focus on ‘positive’ instances where the claimed Franco–German leadership was present, that is, where France–Germany emerged as leaders and made a distinct contribution to crisis resolution. By contrast, scholars have paid less attention to cases where Franco–German leadership did *not* occur in the first place or *failed* to make an impact. This selection corresponds to a certain bias in EU scholarship according to which researchers tend to focus on integration rather than *dis*integration, and on ‘productive’ crises leading to more integration rather than ‘*un*productive’ crises which do not (Gilbert 2008; see also Schoeller 2018).

To address such shortcomings in the existing literature, this article tests and compares the potential for Franco–German EU leadership during crises and the different ways and means this bilateral leadership takes. Furthermore, the article consciously includes instances where Franco–German leadership failed or did not occur in the first place. Case selection thus is motivated by instances of crises rather than any particular form of Franco–German action. Drawing from authoritative accounts of European integration history (e.g., Dinan 2014; Loth 2015; Gilbert 2021), the EU until recently arguably faced nine instances of major crises. These include the EDC crisis from 1952 to 1954; the empty chair crisis of 1965/66; the oil crisis of 1973/74; the budgetary rebate crisis from 1979 to 1984; the end of the Cold War crisis from 1989 to 1991; the Constitutional Treaty crisis from 2004 to 2007; the Euro crisis from 2010 to 2012; the migration crisis of 2015/16; and the Covid-19 crisis of 2020. These cases have in common that they concerned more than a single member state, a single policy field, or the temporary delay in the implementation of EU decisions. Instead, all crises threatened or called into question key principles, features, or objectives of European integration. Moreover, the selection of cases ensures variation over time and thus both in EU membership and the development of international context.

Drawing from established demand–supply models in EU scholarship, international relations, and public policy (Majone 1996; Mattli 1999; Genschel and Jachtenfuchs 2014; Schoeller 2019), the article argues that France and Germany emerge as (potential) leaders in crises and *supply* leadership if they consider status quo costs to have become prohibitively high. This usually is the case when the further existence of the EU or the European integration process is at stake. France–Germany are particularly likely to supply leadership if they themselves are strongly affected by a crisis. Moreover, France–Germany are likely to exercise a decisive impact on EU crisis management if other member states *demand*, or at least tolerate, bilateral leadership. This tends to be the case in the event of high status quo costs for the other member states and if France and Germany have available indispensable leadership resources that others rely on. In addition, France–Germany themselves might create (additional) demand for bilateral leadership if they change the status quo for other member states through bilateral initiatives (Gruber 2000).

As argued above, leadership implies the definition and realization of common policy objectives. With respect to European integration crises, successful leadership means stabilizing the regional polity, further integrating supranational competences and policy fields, and realizing planned integration projects. I expect leadership to be successful if France–Germany emerge as leaders due to high status quo costs; if there is (some) demand for leadership on the part of other member states; and if France–Germany make a decisive impact on crisis resolution, either via complementary package deals or convergent policy preferences. By contrast, unsuccessful leadership implies a weakened and more vulnerable EU polity after a crisis as compared to before, manifestations of European disintegration, and/or missing the realization of planned integration projects. Unsuccessful leadership essentially finds two expressions: first, in the event of absent bilateral leadership, the potential leaders are prevented from emerging as such. This might be due to domestic European treaty ratification problems. Second, next to weak demand on the part of other member states, failed bilateral leadership is the result of France and Germany lacking a common objective for crisis resolution and/or holding irreconcilable policy positions.

For the assumptions about the emergence and impact of Franco–German leadership during EU crises to hold, one must find certain indicators and mechanisms in the analysis. Such “empirical manifestations” (Beach and Pedersen 2019) include the availability and use of bilateral resources; the call and expectations of other member states for France and Germany to play a prominent role in the crisis at hand; intensified bilateral coordination, both at the administrative and the highest political level; the congruence of EU crisis resolution with prior Franco–German proposals; the presence of at least some ways and means of bilateral leadership suggested above; and a secondary role played by other member states and the EU’s supranational institutions. In terms of data, the article reconstructs historical events on the basis of different primary sources including archival material, official EU and national policy documents, memoirs by leading policymakers, and expert interviews with EU and national officials. It triangulates, compares, and complements these sources with secondary literature for each crisis.

## Successful Franco–German crisis management

Ways of successful Franco–German EU crisis management comprise, first, diplomatic brokerage. This implies that France and Germany, at first, represent different or even opposed preferences and different larger camps of member states. Through diplomatic coordination, usually at the highest political and administrative levels, both countries manage to align, via a “compromise by proxy” logic (Koopmann 2004), other member states behind their suggested bilateral policy proposals. A second way of successful crisis management includes France and Germany carrying a disproportionate share of financial burdens to enable solution for a given problem. Thanks to their size and financial resources—as expressed, for instance, in the two countries’ contributions to the EU budget—Franco–German consent is a necessary condition for every EU-level action requiring large amounts of money. Both diplomatic brokerage and financial burden-carrying can happen via complementary package deals following an additive logic or, alternatively, via joint and convergent bilateral policy proposals (Schild 2013). Cases of successful Franco–German EU crisis management comprise the empty chair crisis, the budgetary rebate crisis, the end of the Cold war crisis, the Euro crisis, and the Covid-19 crisis. The following sections consider each crisis in turn.

### Empty chair crisis

In the mid-1960s, the French government, in the person of President Charles de Gaulle, sought preventing an EEC with more autonomous decision-making procedures and a stronger role for supranational institutions, notably the European Commission. Concretely, de Gaulle opposed the scheduled transition to majority voting in the Council of Ministers and additional Commission competences (van Middelaar 2008). In late June 1965, he withdrew his ministers from all Council formations and paralyzed EEC policymaking. Germany criticized France’s behavior and rejected formal changes to the Community’s contractual basis. While Belgium and Luxembourg, at first, wanted to accommodate France’s calls, Germany, together with the Netherlands, was determined to preserve the substance and wording of the Treaty of Rome, the EEC’s founding document. Due to its political and economic weight in Community decision-making and its special relationship with France—documented in the signing of the Franco–German ‘Elysée’ treaty of friendship two years before the crisis erupted—the German government became the main representative of the camp of member states that rejected any formal change to the Rome Treaty. The Italian EEC Council Presidency at the time even explicitly encouraged Germany to play a mediating role and explore options for compromise with France. Thus, while official decision-making at the EU level was blocked, France and Germany, thanks to the Elysée framework, kept bilateral, diplomatic communication channels open (Varsori 1991).

In view of the high political importance that they attached to continued work as the Community of the Six, German policymakers and civil servants preferred a revised EEC, which would accept some of de Gaulle’s wishes, over both a purely European economic organization with no supranational elements at all or, alternatively, a political organization without France (Bajon 2010: 257–270). At the same time, Germany was eager to preserve the Community’s founding principles, such as the possibility of majority-voting. In late October 1965, Germany, together with the other four governments, signed a secret document excluding any formal change to the Rome Treaty. France, in turn, insisted on the maintenance of a national right of veto. However, not least due to its high economic dependence and profits from the EEC, the emerging European common market, and notably the Common Agricultural Policy (Palayret 2006: 70), the French government signaled the option for an interpretative protocol clarifying the practical use of the majority principle.

Coming together in Luxembourg on two occasions in January 1966, to the exclusion of the Commission, delegations of the six member states put an end to the crisis and resumed normal Council working. The Luxembourg compromise stipulates that in the event of disagreement, and irrespective of formal voting rules, national delegations would continue negotiating until consensus was reached (EEC 1966). As an ultimate means, however, as the Rome Treaty foresaw, majority voting would remain possible. Member states also curbed the Commission’s authority with respect to legislative initiative and external representation without, however, touching its formal competences. Both France and Germany, the latter acting on behalf of the Five, had gotten their way, which became visible when all parties claimed victory following the Luxembourg meeting.

### Budgetary rebate crisis

Shortly after having come to power in 1979, the British Prime Minister, Margaret Thatcher, called into question the financing of the Community budget. As a member state that had only joined in the early 1970s when key features and principles of the EEC already were in place, and due to the structure of its agricultural and trading sectors, the United Kingdom (UK) received relatively few subsidies from the Common Agricultural Policy, while transferring large sums of agricultural levies and import duties from trade with Commonwealth countries to the Community. Despite being one of the poorest member states at the time, the UK was thus a major ‘net contributor’ to the EEC budget, second only to Germany (Denton 1984). Asking for a general and permanent reduction to the British payments and—in an attempt to improve its bargaining position—vetoing any increase of the Community’s income ceiling, Thatcher threatened the sustainability of EEC finances, especially in view of the upcoming enlargement rounds. More than any other country, France opposed calls for national rebates and insisted that levies and customs were Community ‘own resources’.

During the early 1980s, national governments on several occasions had negotiated temporary reductions to the British payments. Chairing the European Council in the first half of 1984, however, France’s President, François Mitterrand, was determined to find a lasting solution to the British budgetary question. Speaking to the European Parliament in May that year, Mitterrand warned that France was prepared to “embark[ing] on fresh initiatives”, including a new European treaty, and to move forward “in a Europe of different speeds” if the UK was not to compromise (EC 1984; see also Pedersen 1998: 92–98). Some weeks earlier, Thatcher had rejected a proposal for an annual British reduction worth one billion ECU (the European Currency Unit). Although supporting general British calls for cuts to Community expenditures, Germany’s Chancellor, Helmut Kohl, at important moments sided with France, seconding that conditions were given to move on in a “two-speed Europe”, if necessary, without the UK. This was an exemplary instance of France and Germany, despite national (financial) preferences actually closer to those of other member states, still joining forces to overcome a major crisis (see also Taylor 1989: 7). The instance also provides evidence for Gruber’s (2000) notion that France–Germany, acting together and stressing their “go-it-alone” power, can change the status quo for other member states. In the present case, they countered British threats of vetoing the Community’s budget due to unanimity requirements with the prospect of differentiated European integration.

Coming together in Fontainebleau in June 1984, and following bilateral negotiations between British and French civil servants, Thatcher accepted an annual British rebate of one billion ECU. In an important concession, she further agreed that any future British rebate would be calculated on contributions based on the harmonized VAT rate and not, as previously claimed, on agricultural levies and customs duties. The Fontainebleau agreement confirmed that member states would continue to collect the latter two on behalf of the Community (George 1998: 155). Irrespective of its own reduction that it obtained on the British rebate, the German government remained by far the largest contributor to the EEC budget. France, in turn, following the Fontainebleau formula, turned from a net recipient into a net contributor, highlighting the necessity of Franco–German combined financial resources for the agreement found.

### End of Cold War crisis

With popular upheavals starting in several Eastern European countries and the Berlin wall eventually coming down in November 1989, the prospect of German unification suddenly appeared real. Next to a global and internal dimension involving the United States (US) and Soviet Union and the two German states, respectively, the end of the Cold War crisis thus also had an important European dimension. Notwithstanding official support, noted in numerous Community documents, most EEC member states held strong reservations about the notion of a unified, larger, and more powerful Germany. Amongst member states, France was most alarmed by the events due to its geographical proximity and role as the co-leader of European integration. As a victorious power of the Second World War, it had a formal say on German unification. Moreover, unification brough back power-political considerations and risked undermining the traditional parity between France and Germany, both in demographical and political terms. This parity had been a key, although implicit, principle of European integration since the beginning (Pedersen 1998; Schild 2013: 1370). French authorities thus linked their consent to unification to advances in European cooperation, most notably in the form of a European monetary union (EMU). France and Germany traditionally hold different and, at times, opposing preferences with respect to monetary, economic, and fiscal integration (see also below). While other member states like Italy and the Netherlands had reservations too, the UK and its Prime Minister, Thatcher, were most outspoken in their willingness to delay, if not prevent, German unification.

Aware of the suspicion in the other member states, Germany’s Chancellor Kohl still hesitated to make any formal commitment to EMU due to internal opposition in the German finance ministry and reluctant public opinion. When France’s President Mitterrand communicated his determination to establish a timetable for EMU at the next European Council summit, scheduled for 8–9 December 1989 in Strasbourg, Kohl at first rejected any concrete date. At the same time, in view of the tensions and high stakes involved, Franco–German cooperation, both at political and administrative levels, strongly intensified in the run-up to and after the Strasbourg summit. According to Dyson and Featherstone (1999: 758), bilateral consultations and diplomatic brokerage represented an “inner negotiation” within the broader European framework. In addition, Pedersen (1998: 122) noted that while inviting other member states to take part in their effort for further integration, France and Germany signaled “that the two would not let themselves be diverted from carrying the Union forward”.

In return for Kohl’s eventual approval to declare EMU an official and timely objective, European leaders welcomed the prospect of German unification. Consequently, the Strasbourg European Council recognized that “the German people will regain its unity through free self-determination”. Simultaneously, it underlined the commitment “to achieve closer unity and make the [European] Community the focal point for a changing Europe” (EC 1989). European leaders thus established a formal link between German unification and (deeper) European integration. Further underlining Franco–German coordination and leadership, in April 1990 the two countries suggested to the Irish Council Presidency a concrete timetable for the realization of EMU. They could do so because France and Germany each represented a different larger camp of member states with respect to monetary and economic preferences, conditions, and beliefs. Here, the “compromise-by-proxy” logic, as suggested by Koopmann (2004), with other member states (except for the UK and Denmark) being represented in Franco–German compromises, became visible in a particularly clear form. Most scholars agree that in return for France successfully pushing for EMU, which eventually was established in the Maastricht Treaty of December 1991, the German government managed to upload its main preferences regarding the eventual design of the single currency (e.g., Bozo 2009: 328–331).

### Euro crisis

The single currency, established via the Maastricht Treaty, and formally introduced in 1999, came under threat starting from early 2010. Rising interest rates, high public debts, and balance of payment problems brought several member states to the brink of bankruptcy. Greece was the first and most dramatic case, for which other member states over the following years would provide bilateral loans in no less than three rescue packages. France and Germany, as the two largest member states with most financial resources, were in a decisive position as they alone would cover or be liable for half of the packages. Franco–German consent thus was at least a necessary condition for every EU decision requiring large amounts of money (Gocaj and Meunier 2013). In addition, the EU’s other large member states—the UK, Italy, Spain—either were not part of the Eurozone or themselves faced financial problems. At first, France and Germany differed in their takes on the crisis and the policy solutions proposed: while President Nicolas Sarkozy advocated fast action including common fiscal efforts, Chancellor Angela Merkel called for national responsibility and fiscal risk-reduction before any risk-sharing. With the crisis worsening and more member states running into financial difficulties, however, France–Germany joined forces and together provided indispensable leadership. Importantly, France and Germany themselves were (potentially) strongly affected by the crisis given the high degree of economic interdependence inside the Eurozone and the high liabilities of their largest banks. Moreover, the fact that France and Germany represented different larger camps of member states again proved advantageous because it enabled them to upload bilateral compromises to the European level and exercise an impact on EMU governance reform “going even beyond their individual weights as large and powerful member states” (Degner and Leuffen 2019: 90).

Bilateral leadership came via different means (Schild 2013). First, in terms of agenda setting, France–Germany developed joint policy proposals, such as to tighten budgetary rules and create temporary and bilateral rescue packages. These packages were later replaced by a permanent rescue fund, the European Stability Mechanism (ESM). Second, with respect to compromise-building, France–Germany found bilateral agreements before enabling compromises and consensus on the European level. The most prominent example concerns the agreement on the fiscal compact—a reform of EU fiscal rules—in return for an early start of the ESM (Schoeller 2019: 93–117). Finally, when consensus among all member states was not possible, France–Germany pursued forms of differentiated integration with a limited number of member states. This, for instance, happened in the case of the fiscal compact, which the UK and, at first, the Czech Republic did not join.

Altogether, Franco–German leadership transformed the governance structures of the single currency. The greatest threat to the Eurozone abated in late summer 2012 after national governments had agreed on the creation of a European banking union and the President of the European Central Bank (ECB), Mario Draghi, had announced that his institution would do ‘whatever it takes’ to preserve the single currency. Importantly, and irrespective of considerable domestic popular and party-political resistance, especially on the part of Germany, Merkel and France’s new President, François Hollande, publicly endorsed the ECB’s policy. According to the European Council President at the time, Herman Van Rompuy (2014), this Franco–German backing helped calming the financial markets. By late summer 2012, EMU thus displayed both more fiscal risk-sharing between member states *and* tighter national budgetary rules. This was because bilateral crisis management followed a complementary and additive logic, with both France and Germany, together with their like-minded partner states, uploading key policy preferences (Schild 2013).

### Covid-19 crisis

When a novel coronavirus spread rapidly across Europe starting from February 2020, it threatened several EU policies simultaneously. First, in view of their different fiscal means to counter the historic recession, interest rates between member states diverged, putting the single currency (again) under stress. Second, to stop or at least delay the spread of the virus, national governments closed borders, thereby undermining the principle of borderless traveling inside the Schengen area. Most importantly, the combination of diverse and insufficient fiscal capacities, closed borders, and disrupted economic supply chains, both globally and in Europe, threatened the integrity of the European single market. To cushion the most immediate damages, the EU’s supranational actors, notably the Commission and the ECB, had taken fast measures, such as suspending fiscal deficit rules and buying (additional) government bonds. To enable strong and even recovery across Europe, however, and to foster political unity, national governments had to step in Krotz and Schramm (2022).

At first, member states differed not only in their individual affectedness by the crisis (in terms of death rates and fiscal resources), but also with respect to their policy proposals: while some member states primarily from the EU’s South, such as France, called for ‘corona bonds’ and the joint issuing of government debt, other Northern countries like Germany advocated using existing instruments and opposed the mutualization of debt. On 18 May 2020, however, following intensified bilateral coordination, Chancellor Merkel and France’s President Emmanuel Macron proposed a temporary European recovery instrument worth €500 billion (Elysée 2020). To finance the instrument, the Commission was to raise money in the financial markets, backed by member-state guarantees via the EU budget. Thus, following the convergence perspective, and other than the analysis of the Euro crisis has shown, the two countries agreed on a single policy instrument, which they then sought uploading to the European level. While Germany had given up its traditional opposition to common debt, France no longer insisted on a permanent instrument and the inclusion of existing debt. The fairly symmetric nature of the Covid-19 crisis, affecting France and Germany to similar extents, facilitated this convergence and fast agreement on a joint policy instrument.

According to civil servants closely involved, the scope and substance of the European recovery plan was developed in bilateral negotiations and in close cooperation with the Commission President, Ursula von der Leyen (author’s interviews with civil servants from France, Germany, and the European Commission; autumn 2020). At the European Council summit on 17–21 July, France–Germany held their lines against the (initial) opposition of several groups of member states: on the one hand, ‘frugal’ countries had criticized the grant component in the proposal and insisted on a larger share of loans. On the other hand, Hungary and Poland sought to prevent stricter supranational budgetary surveillance and proposed formulations on the rule of law. However, with France–Germany signaling that they were ready, as an ultimate means, to realize the recovery plan on an intergovernmental basis with like-minded member states, Hungary and Poland lifted their resistance so that national leaders endorsed the recovery plan in December 2020 (Fleming and Khan 2020).

## Unsuccessful Franco–German crisis management

There can be different reasons why Franco–German EU crisis management fails or does not emerge in the first place. In addition to limited demand for bilateral leadership on the part of other member states, one reason is that at least one of the two partners faces major obstacles in the domestic political arena with respect to EU-level measures. Franco–German relations themselves might be at the core of the crisis, with mutual suspicion preventing bilateral leadership from emerging. Another reason is the lack of common purpose and objectives between France and Germany regarding crisis resolution. As a consequence, French and German positions on a given crisis might turn out to be irreconcilable so that attempts at bilateral leadership fail. Instances of unsuccessful Franco–German crisis management include the EDC crisis, the oil crisis, the Constitutional Treaty crisis, and the migration crisis.

### EDC crisis

When North Korean forces invaded the South in May 1950, the US called on Western European countries to provide military support to fight back communism in the looming Cold War. Crucially, US calls included (West) Germany and the restoration of a German army (Fursdon 1980). Just five years after the Second World War, with Germany still being an occupied country, the prospect of German military forces caused resistance among neighboring countries, most notably in France. Taking the initiative and preempting stronger US pressures, on 24 October 1950, France’s Prime Minister, René Pleven, proposed the creation of a European army with a European defense minister. Importantly, the Pleven plan discriminated against Germany in that it sought to prevent the creation of an independent German army and military staff. The French government also wanted to ensure that its own forces would always equal or exceed German ones. In May 1952, six European countries—the same ones which a year earlier hat put in place the European Coal and Steel Community—signed the EDC Treaty. With discriminatory measures largely removed, the EDC foresaw supranational command structures and the “full fusion” of national military forces (CED 1952).

Ratification of the EDC Treaty, however, proved difficult. Smaller member states like Belgium and the Netherlands were reluctant to give up national military command. The German government considered direct entry into the US-sponsored North Atlantic Treaty Organization (NATO) the fastest and safest way to restore national sovereignty. Demonstrating his European commitment, however, Germany’s Chancellor, Konrad Adenauer, still advocated the European army. By spring 1954, four member states had ratified the EDC Treaty, with Italy close to do so. Most opposition came from France. Between March 1952 and June 1954, three governments collapsed mostly due to divisions or lacking parliamentary support for the European army (Lerner and Aron 1957). It became obvious that the EDC proposal from the beginning had not been a French preference but was largely the reaction to the US’ calls for German armament. Subsequent French prime ministers wanted to renegotiate the terms of the EDC Treaty, removing its supranational character and re-introducing discriminatory elements against Germany. When, in August 1954, the other member states rejected further renegotiations, the European army definitely failed obtaining the support of the French parliament.

Like in the other crises under consideration, France and Germany again were the most important actors. With the French EDC proposal primarily concerning military manpower, both countries would provide most troops for any European army. However, still lacking national sovereignty and political authority, Germany was not able (yet) to play a prominent role in European politics, let alone in military affairs. In turn, ratification problems in France proved too strong. With French domestic politics paralyzed, no other signatory country could step in. Mutual suspicion and irreconcilable national interests gave little room for Franco–German leadership. For both countries, the EDC actually had been a means to another higher end (Fursdon 1980: 341): while France sought imposing permanent constraints on German military capabilities, Germany’s priority was to regain national sovereignty. As a result, the initiative moved to a ‘third’ country, the UK, which promoted the creation of the Western European Union (WEU). Other than the EDC, the WEU was a loosely integrated, intergovernmental organization under the primacy of NATO and the US. Western (continental) European countries, however, proved unable to build a supranational defense system for themselves, by themselves.

### Oil crisis

Following the outbreak of the October ‘Yom Kippur’ war in 1973, the Organization of Arab Petroleum Exporting Countries (OAPEC) restricted the production and export of oil to ‘Western’ industrialized countries considered to support Israel. In early November, OAPEC announced immediate cutbacks of 25 percent compared to September levels, to be followed by further cutbacks of five percent every month until Israel withdrew to its former borders. Seeking to divide EEC member states (Möckli 2009: 189–191), OAPEC differentiated between “friendly” countries like France and the UK, that continued receiving their normal oil shares; “unfriendly” countries like Germany, that experienced targeted import restrictions; and “hostile” countries openly supporting Israel, such as the Netherlands (and the US), that were subject to a full oil embargo. Subsequently, the Dutch government urged other member states to pool Europe’s oil reserves and share them, if necessary (Secrétariat Général 1973). The European Commission, noting its competences in matters related to the common market, called for European solidarity and the establishment of an oil-sharing mechanism. National governments, for their part, in previous years had stressed their commitment for a common energy policy and to speak with a single voice in international affairs.

During the oil crisis, however, EEC member states failed to reach a common response. Crucially, Franco–German divisions were at the core of the European divide, leading Haig Simonian (1985: 193–218) to conclude that the bilateral relationship had reached a historical low point. France and Germany differed with respect to both European energy and foreign policy: on the one hand, France, together with the UK, negotiated preferential agreements with Arab countries to secure their own oil deliveries in return for economic investment. It also advocated for a distinct European foreign policy and direct negotiations between European consumer and Arab producer countries. On the other hand, Germany suggested the sharing of energy among Community member states. Regarding foreign policy, it accepted subordination to US hegemony and, at the Washington Energy Conference in February 1974, favored proposals to establish a transatlantic oil-consumer cartel. According to his French counterpart, Germany’s Finance Minister, Helmut Schmidt, declared that “[i]f I must choose between Europe and the United States, I choose the United States, let me be clear about this” (Jobert 1976: 380; my translation).

A further expression of lacking or very limited internal European cooperation was that member states even restricted the export of oil among each other. This not only went against EEC primary law and the principle of the free movement of goods, but it also risked undermining Europe’s common market. Concerning the external dimension of the crisis, in November 1974, eight of the nine EEC member states joined the US-sponsored International Energy Agency. With France refusing to join, and the others rejecting alternative French proposals for a European energy agency, the European split over energy and foreign policy was perfect (Keohane 1984: 220–222). This split, both at the European and the bilateral level, was primarily because France and Germany held different national preferences and priorities, which turned out to be irreconcilable.

### Constitutional treaty crisis

After three European treaty revision rounds in less than ten years with small adjustments that were largely considered insufficient, policymakers in the early 2000s sought putting the EU on a new legal and political basis. In May 2000, the German Foreign Minister, Joschka Fischer (2000), had suggested the EU to move into a “lean federation”, based on a formal constitution and through full parliamentarization. Over a one and a half-year period, a European Convention, starting in February 2002 and composed of policymakers and civil-society representatives, developed institutional and policy proposals. In summer 2003, the Convention presented its ‘Draft Treaty establishing a Constitution for Europe’. Although national governments, in the subsequent intergovernmental conference, altered and limited some of the more ambitious proposals, the Constitutional Treaty still kept most of the provisions that the Convention had suggested, such as genuine EU laws, more powers for the European Parliament, and the post of an EU foreign minister. According to one analyst, the Constitutional Treaty has been the most ambitious project to date to bring the EU closer to its citizens and to publicly legitimize, ex post, a Union founded and largely driven by elites (Phinnemore 2013: 3–4).

To enter into force, ratification in every member state was necessary. Most member states did so via parliamentary approval. Some governments, however, opted for a national referendum. In April 2004, the British Prime Minister, Tony Blair, had declared that he would put the Constitutional Treaty to popular vote. This move put pressure on other national leaders to follow the example. Confident that he would obtain a solid majority, and seeking to split the political opposition at home, President Jacques Chirac announced a French referendum. On 29 May 2005, almost 56 percent of French voters rejected the Treaty. Previous (and later) popular opposition to EU treaties had not dramatically altered the course of European integration. In 1992, for instance, after Danish citizens at first had rejected the Maastricht Treaty, the other member states conceded national ‘opt-outs’ and called for a second Danish vote. In the case of France, however, as a large and founding member state, and with a clear majority in opposition to the Constitutional Treaty, the voting result arguably mattered more (Hainsworth 2006).

Facing domestic ratification problems which, in early 2007, also affected the presidential elections, French policymakers fell out with respect to reviving the constitutional project. Despite its actual support for the Treaty, the German government opted for an intergovernmental conference only and little popular scrutiny. For Germany, securing a treaty reform at all was more important than adhering to formerly declared democratic principles and the original objective to create a European constitutional document. Under the German EU Council Presidency in the first half of 2007, member states negotiated yet another reform treaty. The Treaty of Lisbon, which entered into force in 2009, removed any state-like symbols and thus failed meeting the initial ambitions of the Constitutional Treaty. Most importantly, following domestic ratification problems, particularly in France, member states abandoned the Convention method. In the end, the Lisbon reform treaty reversed the initial objective of replacing existing treaties by a single EU foundational document and fell short of a “distinctive constitutional character” (Crum 2012: 159).

### Migration crisis

In 2015 and 2016, the EU faced an unprecedented number of asylum seekers coming to Europe, with more than 2.6 million asylum applications being launched during these two years. Strong migratory pressures and little EU-level coordination threatened the Common European Asylum System (CEAS) and the Schengen borderless area (Trauner 2016). Both policy regimes are linked in that an effective control of external borders is a precondition for free internal traveling across member states. Moreover, the ‘Dublin’ rules stipulate which member state is responsible for assessing an asylum application. In practice, this usually have been the member states at the EU’s external borders, mostly Greece and Italy. In return, respective Treaty provisions suggest that the other member states relieve these ‘frontline’ countries financially and through the relocation of recognized refugees.

Despite the record overall numbers, member states were very differently affected by the migratory pressures. In absolute numbers, in 2015 and 2016 Germany received by far the most EU asylum applications; in relative terms, most asylum seekers registered in Hungary, Sweden, Austria, and Germany. Consequently, incentives for common measures and demand for any leadership promoting the more equal distribution of refugees, were low (Biermann et al. 2019). In September 2015, following German insistence and French support, member states agreed to relocate, within the next two years, 160,000 refugees from Greece and Italy across the EU. Given the resistance of many national governments, the realization of this decision was remarkable and an expression of Franco–German influence (author’s interviews with civil servants from Germany and the Council of the EU; spring 2018). However, in view of the absolute numbers, the relocation mechanism was clearly insufficient. Moreover, France, like most other member states, opposed further proposals to increase the scope of the mechanism and to turn it into a permanent instrument. The asymmetric character of the crisis goes a long way to explain halfhearted Franco–German leadership supply. Indeed, the two countries were affected very differently, with Germany registering four times more asylum applications than France (Tardis 2016).

Lacking allies, in September 2015 the German government introduced national border controls to cope with the migratory pressures. France followed some weeks later. By that time, eight member states had installed border checks, threatening the future of borderless traveling inside the Schengen area. The measures that national governments took usually concerned limiting the number of migrants coming to Europe and externalizing migratory movements to ‘third’ countries, such as Turkey. Genuine European provisions, by contrast, largely remained absent. Instead, largely satisfied with the status quo, a majority of member states did not fulfill their allocated targets from the relocation mechanism. Some member states even questioned the legality of the decisions taken and the authority of the Court of Justice of the EU. Occasional attempts at bilateral leadership, such as for reforms of the CEAS or the Schengen area (Ayrault and Steinmeier 2016), either lacked the support of other member states or were not given further substance by Franco–German policymakers themselves.

## Conclusions

This article has assessed the potential, ways, and means of Franco–German leadership in EU crisis management. As founding and the two largest member states, France and Germany are at the center of European integration and the development of what today is the EU. Remarkably, however, little research exists on bilateral leadership during crises, let alone in a comparative perspective covering the entire integration process and various crises over time. The article suggests that France–Germany are likely to emerge as leaders if they consider status quo costs to have become prohibitively high. This tends to be the case if the further existence of the EU (or its predecessor organizations) is at stake. It is even more likely if France and Germany themselves are strongly affected by the given crisis.

The article has demonstrated the pivotal role that member states play in the management and resolution of major integration crises. Due to the high stakes and great uncertainty involved, crises in particular require actors that are able and willing to take the lead. Leadership implies the stabilization and, at times, further integration of the regional polity. In an EU built on anti-hegemonic principles and characterized by strong heterogeneity in terms of member states’ size, interests and outlooks, leadership usually takes the form of joint or shared leadership. Given their large resources and institutionalized bilateral relationship, France and Germany are the most likely candidates to do so. Interestingly, the potential of Franco–German leadership has remained stable over time, largely irrespective of changes in the (party-political) composition of government in the two countries and in the international and European political context.

At the same time, the emergence and impact of Franco–German EU leadership are crisis-specific. As notably the four case studies on unsuccessful bilateral crisis management have shown, the potential for bilateral leadership is limited if there is little demand for it among other member states due to their satisfaction with the status quo and/or the availability of more promising national alternatives. Moreover, France and Germany are less likely to emerge as leaders if one of the two, or both, face strong domestic obstacles, such as European treaty ratification problems. Finally, France and Germany are unlikely to make a decisive impact on EU crisis politics if the two countries themselves are very differently affected by a crisis and if they do not share a common objective with respect to crisis resolution. Table 1 below gives an overview of the empirical findings from the case studies, distinguishing between demand and supply conditions, ways and means of bilateral leadership, and the resulting crisis outcome.

**Table 1:** Crisis, demand and supply conditions, ways and means of bilateral leadership, crisis outcome

Crisis	Demand	Supply	Ways and means of bilateral leadership	Crisis outcome (with respect to European integration)
EDC	Low: keep national sovereignty over the military; more promising alternative in form of NATO/WEU	Low: not in a position to provide leadership due to lacking authority (GER) and domestic ratification problems (FR)	Unsuccessful, absent leadership due to domestic ratification problems	Failed realization of planned project: WEU (intergovernmental alliance) instead of European army
Empty chair	High: strong political and economic dependence on EEC	Medium: FR and GER represent different ends of spectrum regarding Treaty change; enable political interpretation of majority rule	Successful leadership via diplomatic brokerage	Stabilization of polity: prevention of French exit and of change to EEC founding Treaty, clarification on majority rule
Oil	Medium: occasional calls for oil-sharing, but different affectedness by crisis and different means to cope with it	Low: FR-GER differences on European energy and foreign policy	Unsuccessful, failed leadership due to overall lack of common objective and different priorities	Weakening of polity and partial disintegration in view of disagreements on foreign policy and trade restrictions inside common market
Budgetary rebate	High: credible UK threat to oppose necessary rise in EEC’s income ceiling due to unanimity requirement	High: FR-GER commitment to move on in integration, if necessary, without UK; joint carrying of largest share of UK rebate	Successful leadership via financial burden-carrying	Stabilization of polity: securing of Community’s finances, clarifications on national budgetary contributions
End of Cold War	Medium: no formal means to prevent GER unification, but concerns about return of power politics in Europe	High: FR and GER represent different camps of member states regarding monetary and economic affairs; bilateral agenda-setting	Successful leadership via diplomatic brokerage	Further integration: transfer of monetary sovereignty from national to European level
Constitutional treaty	Low: reservations in several member states; ‘fallback’ option of current Nice Treaty or another European reform treaty	Low: little interest and means to revive constitutional project due to ratification problems (FR) and preference for ‘safe’ reform treaty (GER)	Unsuccessful, absent leadership due to domestic ratification problems	Failed realization of planned project: Lisbon Treaty (another reform treaty) instead of European constitution
Euro	High: strong economic and financial interdependence inside EMU; indispensable FR-GER financial resources	High: FR-GER commitment to deepen EMU; complementary reforms; FR-GER backing of ECB policy	Successful leadership via financial burden-carrying	Further integration: more fiscal risk-sharing, creation of European banking union
Migration	Low: asymmetric crisis with majority of member states hardly affected and/or having more promising alternatives available	Medium: FR-GER receive almost half of all asylum-seekers in EU, push through temporary relocation mechanism; however, few (successful) proposals for thorough reforms due to different affectedness by crisis	Unsuccessful, failed leadership due to overall lack of common objective and different priorities	Weakening of polity and partial disintegration in view of overreliance on third actors (Turkey), introduction of border controls, undermining of EU law
Covid-19	High: triple threat to Eurozone, Schengen area and single market; FR-GER with large financial resources, representing different camps of member states	High: FR-GER blueprint for EU recovery plan; consensus-building via threat to implement recovery plan on intergovernmental basis, if necessary	Successful leadership via financial burden-carrying	Further integration: common issuance of debt, more fiscal risk-sharing

Future research should scrutinize more closely the domestic conditions in France and Germany for the emergence of bilateral leadership. Due to space constraints, this article referred to domestic obstacles primarily in the form of European treaty ratification problems. At the same time, notwithstanding their traditional reluctance towards common debt, German policymakers and voters approved the bilateral initiative for a European recovery plan. Similarly, French governments in recent years have repeatedly promoted, together with Germany, reforms of EU fiscal and economic governance, which large parts of the French political class and society actually oppose. Exercising leadership in and for the EU, France–Germany, at times, develop bilateral policy preferences which deviate from strictly defined national interests. The crucial question is when, how and why domestic politics approves or, alternatively, opposes bilateral coordination at the highest political level.

In addition, scholars should investigate the conditions for support, or at least endorsement, of Franco–German leadership on the part of other member states. During the Covid-19 crisis, ‘frugal’ countries initially opposed the Franco–German initiative before, eventually, joining the European recovery plan. Previous examples of France–Germany signaling ‘go-it-alone’ power and altering the status quo for other member states, thereby prompting them to join bilateral initiatives, include the budgetary rebate crisis, the introduction of the single currency following the end of the Cold War crisis, and the reform of fiscal governance in the context of the Euro crisis. With respect to the recent Russian war against Ukraine, it would be interesting to analyze whether and to what extent primarily Central and Eastern member states, who geographically and historically are much closer to the events, accept potential Franco–German leadership offers. In turn, France and Germany must demonstrate what their contribution to this crisis could be and how Franco–German leadership might look like, also with respect to ‘third’ countries like the US and the UK.

The article has some further, primarily empirical implications. As, for instance, the management of the Euro crisis has shown, Franco–German leadership often follows an additive logic based on complementary package deals through which both countries realize national preferences. This can be problematic in several respects: first, as again the Euro crisis shows, such an approach arguably leads to suboptimal policy outcomes. Following Franco–German leadership, EMU neither developed into a much stricter policy regime based on national responsibility and limited public spending, as favored by Germany, nor into a European fiscal union with common debt instruments, as suggested by France. Second, ambiguous formulations, such as the (European) Council conclusions ending the empty chair crisis and the budgetary rebate crisis, respectively, provide much room for political interpretation and exploitation. Although never formally established, an (implicit) national right of veto remained in place until at least the 1980s. Similarly, no national rebate had been created at the 1984 Fontainebleau summit. Yet, since France and Germany themselves could not agree on concrete policy solutions in both cases, the EU-level crisis ‘resolution’ paved the way for disputes and national challenging in the years to come. Presumably complementary package deals and ambiguous formulations might thus make the EU vulnerable in the longer term and prone to new crises.
